# Nucleoid-Associated Protein HU: A Lilliputian in Gene Regulation of Bacterial Virulence

**DOI:** 10.3389/fcimb.2019.00159

**Published:** 2019-05-10

**Authors:** Pavla Stojkova, Petra Spidlova, Jiri Stulik

**Affiliations:** Department of Molecular Pathology and Biology, Faculty of Military Health Sciences, University of Defence, Hradec Kralove, Czechia

**Keywords:** HU protein, DNA-binding protein, pathogenesis, virulence, nucleoid-associated protein

## Abstract

Nucleoid-associated proteins belong to a group of small but abundant proteins in bacterial cells. These transcription regulators are responsible for many important cellular processes and also are involved in pathogenesis of bacteria. The best-known nucleoid-associated proteins, such as HU, FIS, H-NS, and IHF, are often discussed. The most important findings in research concerning HU protein are described in this mini review. Its roles in DNA compaction, shape modulation, and negative supercoiling induction have been studied intensively. HU protein regulates bacteria survival, growth, SOS response, virulence genes expression, cell division, and many other cell processes. Elucidating the mechanism of HU protein action has been the subject of many research projects. This mini review provides a comprehensive overview of the HU protein.

## Introduction

Elucidating the regulation of bacterial virulence has been a subject of interest for many years. Well-developed mechanisms regulating virulence strategies have emerged from coevolution of pathogens and their hosts. Bacterial pathogenesis is dependent on synthesis of various virulence factors whose production is under specific gene regulation control mediated by various transcription factors. Eukaryotic cells contain small but abundant proteins, known as histones, which are responsible for DNA's compaction into the nucleosome and thus are important for many molecular processes. Prokaryotic cells also contain this abundant group of small proteins which are similar to eukaryotic histones. They are called histone-like or nucleoid-associated proteins, and it is presumed that they fulfill similar biological functions as do their eukaryotic counterparts (Pettijohn, 1988). The former designation “histone-like proteins” is derived from their physical and chemical behavior. For example, they have low molecular weight, high copy number in the cell, and high electrostatic voltage (Bahloul et al., 2001). The more accurate term for this protein group is nucleoid-associated proteins (NAPs), so named for their localization in nucleosomes (Azam and Ishihama, 1999). Due to their influence on important molecular processes, these proteins can play key roles in basic metabolic pathways, stress response, virulence, and interaction with host cells. Among the best known proteins of this group are the HU, FIS (factor for inversion stimulation), H-NS (histone-like nucleoid structuring), and IHF (integration host factor) proteins (Dillon and Dorman, 2010). This review focuses on HU proteins.

## Nucleoid-Associated Protein HU

### Discovery of HU Protein and Its Nomenclature

In 1975, a small, low-molecular-weight DNA-binding protein was isolated from *E. coli* strain U13 (Rouvière-Yaniv and Gros, 1975). At first, it was referred to as Factor U. Due to its properties and characteristics, this protein displayed an interesting resemblance to eukaryotic histones and was denoted as “HU” protein (Rouvière-Yaniv and Gros, 1975; Drlica and Rouviere-Yaniv, 1987). With its DNA-binding ability, HU protein helps in stabilizing nucleic acid against thermal denaturation (Rouvière-Yaniv et al., 1977). Some reports have stated that the designation “HU” stands for “heat unstable,” and many studies have focused on the thermal stability of HU protein. Notably, HU protein from thermophilic bacteria, such as *Spiroplasma melliferum* or *Thermotoga maritima*, have shown high thermal stability (Christodoulou et al., 2003; Boyko et al., 2016). Thermal stabilities of HU protein in other bacteria also have been investigated. The melting point of HU proteins from *E. coli* (Ramstein et al., 2003)*, Bacillus stearothermophilus* (Kawamura et al., 1998), and *B. subtilis* (Welfle et al., 1992) range from 27 to 72°C, depending on their concentrations. Due to the diversity in thermal stability of HU proteins among various bacteria, designations of “heat stable” or “heat unstable” are often used and HU protein terminology is in many cases confusing.

### Protein Structure

HU protein is among the most abundant and conserved nucleoid-associated proteins in eubacteria (Azam and Ishihama, 1999). The greatest abundance of HU protein has been detected during the exponential growth phase, when it reaches 30,000 to 55,000 molecules per cell in *E. coli* (Ali Azam et al., 1999). HU protein exists as a homodimer in most bacteria, but in *Enterobacteriaceae* it forms a heterodimer. In *E. coli*, for example, HU protein forms a heterodimer consisting of two subunits: HU-α and HU-β, encoded by the *hupA* and *hupB* genes (Pettijohn, 1988). The HU protein in *Mycobacterium tuberculosis* is a homodimer consisting of only one subunit: HU-β, encoded by the *hupB* gene (Bhowmick et al., 2014). In the Gram-positive bacterium *Clostridium difficile*, by contrast, only the *hupA* gene has been identified (Oliveira Paiva et al., 2019).

**Figure 1:**
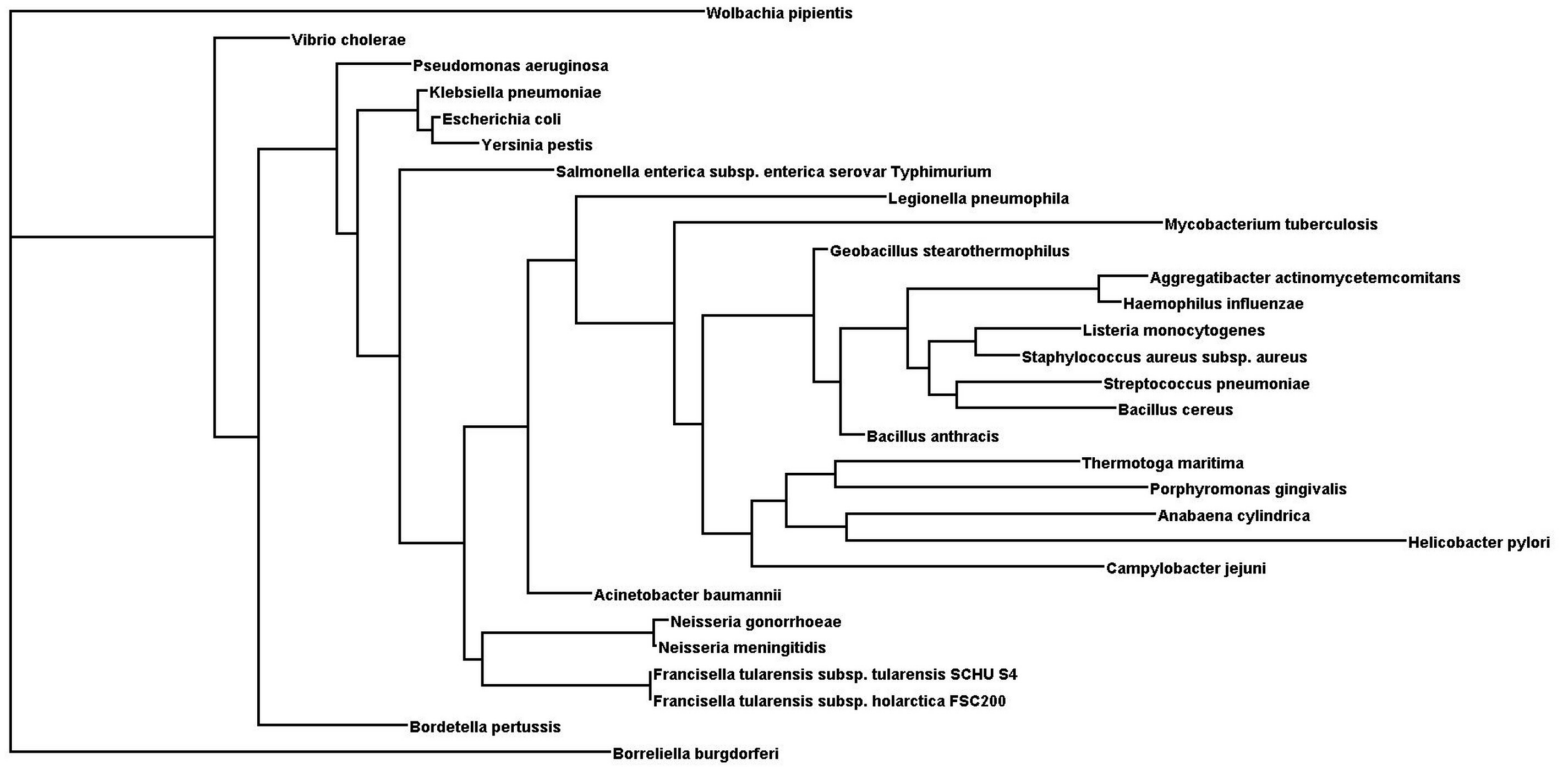
Phylogenetic tree of selected HU proteins. Input file for phylogenetic relationships was generated by Clustal Omega. Phylogenetic tree was generated using the protein Maximum Likelihood method (Proml) in the PHYLIP package (version 3.695). Phylogenetic tree was displayed with Dendroscope (version 3.5.9).

**Figure 2:**
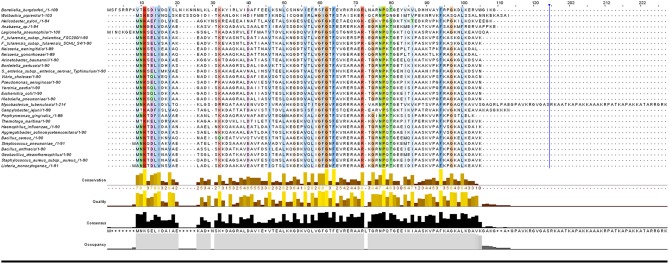
Sequence alignment of selected HUβ proteins. Protein sequences of the 29 strains were aligned with Clustal Omega (version 1.2.4) using default parameters. Alignment results were visualized by Jalview (version 2.10.5).

It has been found that some amino acids are important for HU protein dimer formation. Lys3, for example, is a key residue for salt bridge formation with Asp26 that leads to HU homodimer formation and also affects binding site length. The HU homodimer is able to bind 37 bp, but the Lys3 substitution shortens the binding site to approximately 25 bp, which is inefficient in forming a stable complex with DNA in *B. subtilis* bacteriophage SPO1 (Grove and Saavedra, 2002). Although HU is a strongly conserved protein and almost all HUs are composed of a 90 amino acid core, the N- and C- terminal extensions can differ. Figure 1 shows phylogenetic relationships and Figure 2 sequence alignment for selected HU proteins of well-known pathogenic bacteria. The HU protein from *M. tuberculosis* is distinct and contains a long C- terminal extension that results in a protein composed of 214 amino acids, thereby making it more similar to eukaryotic histones than are others (Ghosh et al., 2016). A recent study by Hołówka et al. (2017) demonstrated that this “eukaryotic-like tail” is indispensable for its *in vivo* association with the nucleoid. Most of the bacterial HU extensions are rich in lysine residues, and “PAKKA” repeats are often present (Kamashev et al., 2017). HU protein structures have been determined in various prokaryotic organisms using different approaches (e.g., nuclear magnetic resonance spectroscopy, X-ray crystallography). HU protein structures have a very common folding that consists of an α-helical part (the “body”) and two β-sheets that are extended to two β-ribbon parts (“arms”) (Christodoulou et al., 2003; Swinger et al., 2003). Studies of *B. stearothermophilus* and *T. maritima* HU protein structures have shown that β-ribbon arms are disorganized in the absence of DNA (Christodoulou and Vorgias, 1998; White et al., 1999), but in the presence of DNA these flexible arms are important for effective binding (Saitoh et al., 1999). Due to its structure, HU protein is able to bind DNA, albeit in a sequence-nonspecific manner (Balandina et al., 2002). Study of *B. stearothermophilus* HU protein has suggested non-specific B-DNA minor groove recognition (Serban et al., 2003). This so-called “shape readout” is based on such aberrations from ideal B-DNA as loops, minor grooves, and intercalation (Kim et al., 2014). β-ribbon arms can induce flexible bends in DNA of 105° up to 180° (Rice et al., 1996; Swinger et al., 2003; Swinger and Rice, 2004; Becker et al., 2005), and proline residues at the tips of the arms are intercalated to the DNA chain (Swinger and Rice, 2007).

### HU–DNA Interaction

HU seems to be an important protein for DNA compaction, replication, transcription, recombination, and shape modulation in many bacteria (Broyles and Pettijohn, 1986; Roy et al., 2005; Oberto et al., 2009). In the coccoid bacterium *Deinococcus radiodurans*, HU protein also plays an important role in nucleoid morphology, and an influence of HU protein on a broad diversity of nucleoid structure has been discovered (Floc'h et al., 2019). HU binds all nucleic acids and their various hybrids in a sequence-non-specific manner (Balandina et al., 2002), but it exhibits high affinity for such abnormal DNA structures as four-way junctions, gaps, or nicks that are generated, for example, during DNA damage (Kamashev and Rouviere-Yaniv, 2000). DNA damage could be generated during oxidative stress conditions by generation of hydroxyl radicals, and this state of affairs occurs in host defense mechanisms. HU protein can help prevent DNA damage through binding to the nucleic acid chain and thus mediate DNA protection (Boubrik and Rouviere-Yaniv, 1995). HU is also able to protect DNA against such intracellular nucleases as exonuclease III (Kamashev and Rouviere-Yaniv, 2000).

HU has the ability to induce negative supercoiling into relaxed DNA molecules in the presence of topoisomerase I and thus influences gene expression (Rouvière-Yaniv et al., 1979). It has also been suggested that HU acts as a functional insulator of transcription units by constraining of DNA supercoiling generated by transcription (Berger et al., 2016). Induction of negative DNA supercoiling by HU protein is dependent upon formation of higher-ordered nucleoprotein complexes, and Glu38, in particular, is necessary. On the contrary, the wild-type strain *E. coli* mutant with HUα^+^β-E38A has shown significantly decreased ability to generate negative DNA supercoiling (Guo and Adhya, 2007). Also, an *E. coli* mutant strain able to introduce positive supercoiling DNA has been isolated. This mutant strain has only two amino acids (HUα E38K and HUα V42L) substituted, and these substitutions lead to cellular changes that result in a shift from commensal to pathogenic mode (Koli et al., 2011).

The non-specific binding sites in double-stranded DNA range in size among the various species. The size of the DNA-binding site is about 9 bp in *E. coli*, (Broyles and Pettijohn, 1986), it is 12 bp in *B. subtilis* (Bonnefoy and Rouvière-Yaniv, 1991), 17–19 bp for *Anabaena* (Lavoie et al., 1996) and *Helicobacter pylori* (Chen et al., 2004), and 37 bp for *B. subtilis* bacteriophage SPO1 (Grove and Saavedra, 2002). Although HU binds DNA in a sequence-nonspecific way, a certain binding motif composed of two DNA helices that can rotate without constraint has been suggested (Kamashev et al., 1999). HU arms interact with the 3′ DNA branch, whereas the HU body interacts with the curved 5′ DNA branch (Kamashev and Rouviere-Yaniv, 2000). *Anabaena* HU binds DNA through a proline residue (Pro63) that is located at the end of the HU β-ribbon arms and is incorporated into the DNA minor groove (Swinger et al., 2003). Many of HU's amino acids have been reported as important for its DNA-binding ability. Arg55, in particular, has shown great importance for DNA-binding activity in *B. stearothermophilus* (Saitoh et al., 1999). Surface-exposed lysine residues play a role in DNA wrapping, length of the binding site, and homodimer formation (Grove and Saavedra, 2002). Particularly noteworthy is that HU dimers are able to bind DNA through locking a DNA phosphate between Gly46 and Lys83 while inserting Val45 into the minor groove such that Val45 acts as a turning point. The importance of these amino acids in DNA binding is also supported by their conservation in prokaryotes (Swinger and Rice, 2004). Additional amino acid residues are reported to be important for interaction with DNA, including Arg53, 55, 58, 61, 64, 80, and Lys86 (Bhowmick et al., 2014). Although HU protein does not recognize specific sequences, it does prefer A/T-rich DNA regions, including pathogenicity island encoding virulence factors (Krylov et al., 2001; Dorman, 2014). Prieto et al. (2012) focused their work on HU and IHF (HU homolog) regulatory networks and their binding motifs. Although they showed different binding motifs for HUα and HUβ in *E. coli*, they concluded that the motifs identification was not reliable and confirmed that HU protein binds in a non-specific manner to DNA, albeit with a preference for DNA having A/T-rich regions.

### HU–RNA Interaction

HU is able to bind all forms of nucleic acids, including DNA–RNA hybrids (Balandina et al., 2002). Although HU has no specific binding motif, it is able to bind mRNA of RpoS (Balandina et al., 2001). Another study of the *B. subtilis* HU protein showed that HU binds an Alu domain of cytoplasmic RNA (Nakamura et al., 1999). HU also recognizes RNA of DsrA that modulates the translation of key cell regulators, such as RpoA or H-NS (Majdalani et al., 1998; Lease and Belfort, 2000). HU actually has shown higher affinity for DNA–RNA hybrids than for RNA or DNA duplexes (Balandina et al., 2002).

### HU–Protein Interaction

Interaction of HU protein at protein level has not yet been fully explored. As mentioned above, an HU protein is able to react with another HU protein to form a dimer together (White et al., 1999; Swinger et al., 2003). Glu38, in particular, is a key amino acid for interdimeric interface in the left-handed spiral formation (Guo and Adhya, 2007). HU can modulate formation of the pre-replication complex of IHF/DnaA/*oriC*, where HU serves as an activator or suppressor, depending upon its concentration (Bonnefoy and Rouvière-Yaniv, 1992; Ryan et al., 2002). HU controls the DNA-multiprotein complex formation, termed repressosome, that regulates transcription initiation of the *gal* operon in *E. coli*. Three models of this interaction have been shown. First, due to two GalR dimers that bend DNA, HU dimer is able to bind and stabilize the interaction between DNA-bound GalR dimers (Kar and Adhya, 2001). The second model suggests an interaction between HU and GalR that results in a complex that is moved to the target binding site. The third model of interaction between HU and GalR presumes binding of HU protein to DNA while GalR dimer remains bound (Geanacopoulos et al., 2001). Three HU amino acids in particular (Ser17, Lys18, and Thr19) seem to be crucial for the HU/GalR/*gal* interaction (Kar and Adhya, 2001). Moreover, the Lys18 has been shown to be acetylated, thus suggesting involvement of HU acetylation in protein–protein interaction (Weinert et al., 2013a). Although HU protein has no sequence specificity, it could play an eminent role in the formation of higher protein complexes that regulate initiation of gene transcription. A direct interaction of HU protein with host protein has been reported in the case of *Aggregatibacter actinomycetemcomitans*, a causative agent for periodontitis. Interaction of HU and interleukin-1beta (IL-1β), an important proinflammatory cytokine modulating anti-pathogen response, was confirmed using two different proteomic approaches (Paino et al., 2012).

### Metabolism and Virulence Association

The function of HU protein as a DNA-binding transcription factor indicates its influence on important metabolic cycles. It can also be presumed to be involved in virulence gene expression in the case of pathogenic bacteria (Mangan et al., 2011; Stojkova et al., 2018). HU protein plays an important role in the initiation of DNA replication (Bonnefoy and Rouvière-Yaniv, 1992), cell division, SOS response (Preobrajenskaya et al., 1994; Oberto et al., 2009), and galactose metabolism (Aki et al., 1996). In *Streptococcus pneumoniae*, HU protein also is essential for cell viability (Ferrándiz et al., 2018). In *E. coli*, HU protein controls 8% of genes across the whole genome. These are associated with adaptation to the unfriendly environment of the host cell or with stress response (Oberto et al., 2009). HU is able to displace the repressor of the SOS response genes, LexA, and thus probably initiates transcription of SOS genes (Preobrajenskaya et al., 1994). Strains lacking HU protein are sensitive to γ and UV irradiation (Boubrik and Rouviere-Yaniv, 1995; Li and Waters, 1998). Noteworthy in the context of *M. tuberculosis* is that this protein is a potential target for the development of therapies against tuberculosis (Bhowmick et al., 2014). Influence of HU protein on the expression of virulence genes has been reported in *Salmonella enterica* serovar Typhimurium (Mangan et al., 2011), *Francisella tularensis* (Stojkova et al., 2018), and *Porphyromonas gingivalis* (Priyadarshini et al., 2013). Deletion of both HU subunits led to a reduction in the growth rate and type III secretion system-related genes were reduced in *Vibrio parahaemolyticus* (Phan et al., 2015). HU likely affects the motility of *Salmonella*, because genes coding for the flagellum showed altered expression in *hup* mutants (Mangan et al., 2011). Similarly in *Xanthomonas citri*, HU protein negatively affects flagellum-associated gene expression and subsequent loss of flagellum has been confirmed by electron microscopy (Conforte et al., 2018). HU protein was shown to be important for cell motility in *Cytophaga hutchinsonii*, whose “non-flagellar” motion is not yet fully understood (Guan et al., 2018). HU also controls transcription of a gene required for anaerobic respiration (nitrate reductase A *narH*). In the case of *E. coli*, HU has been reported to regulate *narH* positively (Oberto et al., 2009). That is in contrast to how HU protein diminishes *narH* expression in *Salmonella* (Mangan et al., 2011). As mentioned, this nucleoid-associated protein is able to play the co-factor role in repression of *gal* transcription and thus has significant effect on energy metabolism in *E. coli* (Aki et al., 1996) and *Salmonella* (Mangan et al., 2011). Enzymes important for oxidative stress reaction are regulated by sigma factor 70 (*rpo*S), and this gene is induced by HU protein (Balandina et al., 2001) This finding is corroborated by a publication showing that an *E. coli* strain lacking the HU protein was unable to respond appropriately to oxidative stress. A similar result was obtained in the case of *Salmonella*, where RpoS protein expression was reduced by inactivation of both *hup* genes (Mangan et al., 2011). HU protein plays an essential role in biofilm formation and overall pathogenesis in *X. citri* (Conforte et al., 2018) and *C. hutchinsonii* (Guan et al., 2018). HU protein seems to regulate a large number of genes and metabolic pathways within the bacterial cell. Its broad field of action explains the diversity in phenotype among strains lacking HU protein. Questions remain as to exactly what processes HU regulates, and whether it does so directly or indirectly.

### Post-translational Modification

Post-translational modifications are very important processes in signal metabolic pathways within cells. Transcription factors, such as nucleoid-associated proteins, are at the beginning of intracellular communication and their modifications are a necessary part of the whole process. HU protein could act as a signal molecule leading to adaptive changes, because it is a target for phosphorylation by serine/threonine kinases in *M. tuberculosis*. Although phosphorylation of HU protein negatively modulates its ability to bind DNA, the necessity of Thr65 and Thr74 for DNA-binding capacity has been described in *M. tuberculosis* (Gupta et al., 2014). Its acetylation at various lysine residues also alters modulation of DNA compaction and ability for DNA binding of HU protein. Acetylation of HU leads to decrease of its affinity for DNA in *Mycobacterium* (Ghosh et al., 2016). *Mycobacterium* has also developed a deacetylation mechanism for acetylated HU protein, and thus it regulates the binding capacity of HU (Anand et al., 2017). Acetylation of mycobacterial HU protein has been found at Lys3, 72, 86, 103, 116, 133, 146, and 167 (Ghosh et al., 2016), and another study (Weinert et al., 2013b) complemented these data on Lys18, 70, and 94 as well as Arg53 and 54 or 55. HU is also subject to methylation (Lys3, 86, 94, 103 and Arg53, 54, 55) (Sakatos et al., 2018). In addition, *E. coli* HUα can be succinylated at Lys86 (Weinert et al., 2013b). There is strong need for further studies focused on the roles of HU's post-translational modifications in its interaction with proteins and nucleic acids.

## Conclusion

The importance of HU protein in bacterial metabolic cell cycles has been established at the DNA, RNA, and protein levels. Its interaction with DNA has long been known, and recently its interactions with other nucleic acids, RNA, and various nucleic acid hybrids have been described. An area that is little researched to date but already recognized for its importance is that of interactions between HU and other proteins. A recently described interaction with the host protein during infection suggests a possible role of the HU protein in regulating host response to infection. More detailed study of post-translation modification could help to clarify mechanisms of its interactions and function in bacterial and/or host cells. Although HU is a small DNA-binding protein, it has wide a variety of roles. HU protein's mode of action seems to be dependent upon conditions of the environment and bacterial life cycle. Its initial control of DNA topology, expression of important metabolic products, and control of virulence gene expression lead to global regulation of bacteria viability. HU protein and its role in DNA compaction and topology have been intensively studied for decades, but the evidence of HU protein secretion and its direct interaction with host cell constitute a noteworthy and so far not well-researched area. HU protein is a well-known transcription factor, but perhaps equal attention should be given to its role as a signal molecule or host antimicrobial response modulating protein. This Lilliputian among proteins has drawn great attention in recent studies of bacterial pathogenicity, and it certainly will be the subject of many future studies concerning virulent bacteria.

## Author Contributions

All authors wrote the paper, reviewed, and approved the manuscript.

### Conflict of Interest Statement

The authors declare that the research was conducted in the absence of any commercial or financial relationships that could be construed as a potential conflict of interest.
